# An Undiagnosed Case of Hypothalamic Hamartoma with a Rare Presentation

**DOI:** 10.1155/2017/2432315

**Published:** 2017-01-26

**Authors:** Shervin Badihian, Saeideh Bahrani, Nasim Tabrizi, Houshang Moein, Mohammad Zare, Majid Barekatain, Reza Basiratnia, Elham Rahimian, Amirali Mehvari Habibabadi, Payam Moein, Jafar Mehvari Habibabadi

**Affiliations:** ^1^Isfahan Neurosciences Research Center, Department of Neurology, School of Medicine, Isfahan University of Medical Sciences, Isfahan, Iran; ^2^Department of Neurology, Mazandaran University of Medical Sciences, Mazandaran, Iran; ^3^Department of Neurosurgery, School of Medicine, Isfahan University of Medical Sciences, Isfahan, Iran; ^4^Kashani Comprehensive Epilepsy Center, Kashani Hospital, School of Medicine, Isfahan University of Medical Sciences, Isfahan, Iran; ^5^Psychosomatic Research Center, Department of Psychiatry, School of Medicine, Isfahan University of Medical Sciences, Isfahan, Iran; ^6^Department of Radiology, School of Medicine, Isfahan University of Medical Sciences, Isfahan, Iran; ^7^Shefa Neuroscience Research Center, Tehran, Iran; ^8^Students' Research Center, School of Medicine, Shahrekord University of Medical Sciences, Shahrekord, Iran; ^9^Department of Neurology, University of Tennessee Health Science Center, Memphis, TN, USA

## Abstract

*Background*. Hypothalamic hamartomas (HHs) are rare tumor-like malformations that may present with complex partial seizures refractory to anticonvulsants in adulthood. The condition may be misdiagnosed because of rarity.* Case Presentation*. We report a 25-year-old man with complaint of seizures presented by falling, tonic spasm of limbs, oral automatism, vocalization, and hypermotor activities. His seizures started at the age of one month and presented as eye deviation and upper limbs myoclonic jerk, followed by frequent seizures with variable frequency. The patient had delayed developmental milestones and was mentally retarded. He was hospitalized and underwent video-EEG monitoring and neuroimaging, and the diagnosis of HH was made. The patient became candidate for surgery after that.* Conclusion*. In this case, the underlying etiology of seizures was diagnosed after 25 years. HH is a rare condition and neurologists may encounter very small number of these cases during their practice. Therefore, they should consider it in patients who present with suspected signs and symptoms.

## 1. Introduction

Hypothalamic hamartomas (HHs) are rare tumor-like malformations, originating from tuber cinereum and inferior hypothalamus [1]. In children, it is classically presented by a triad including gelastic seizures, developmental delay, and precocious puberty [2]. In adult patients gelastic epilepsy is uncommon and they tend to develop complex partial seizures with or without secondary generalization [3]. Seizure semiology shows laughing and multiple other seizure types. It most often leads to severe drug resistant epilepsy [4]. In this paper we report a patient experiencing complex partial seizures and tonic seizures secondary to hypothalamic hamartoma displacing mammillary body. It must be noted that the patient gave consent to the anonymous publication of his data.

## 2. Case Presentation

A 25-year-old right handed man was admitted to Kashani comprehensive epilepsy center due to drug resistant epilepsy for video-EEG monitoring. He had complaint of seizures presented by falling, tonic spasm of limbs, oral automatism, vocalization, and hypermotor activities. He also reported experiencing light flashing as aura sometimes although the auras were not repeated during the hospitalization in our center. Neither jaw locking, hemiparesis, postictal state, headache, vertigo, periaural numbness, nor dysarthria was reported. In his past medical history, he was born through cesarean section (because of breech presentation) with no history of perinatal complications or head trauma. He experienced first episode of afebrile seizure at the age of 1 month presented as eye deviation and upper limbs myoclonic jerk. After that, he suffered from frequent seizures with variable frequency starting at 6 months of age. The pattern of seizures switched to current one few years ago. His developmental milestones were abnormal and delayed. He also had documented vitamin D insufficiency which was substituted at time of diagnosis. There was no history of seizure disorders or any other neurological disease in his family. His epilepsy was managed by anticonvulsants including Levetiracetam 1000 mg/day, Primidone 1050 mg/day, and Lamotrigine 200 mg/day although the medical therapy was not successful in discontinuation of seizures. No underlying reason was diagnosed for his seizures before. Although neuroimaging was performed before, no diagnosis was determined for the patient.

**Figure 1:**
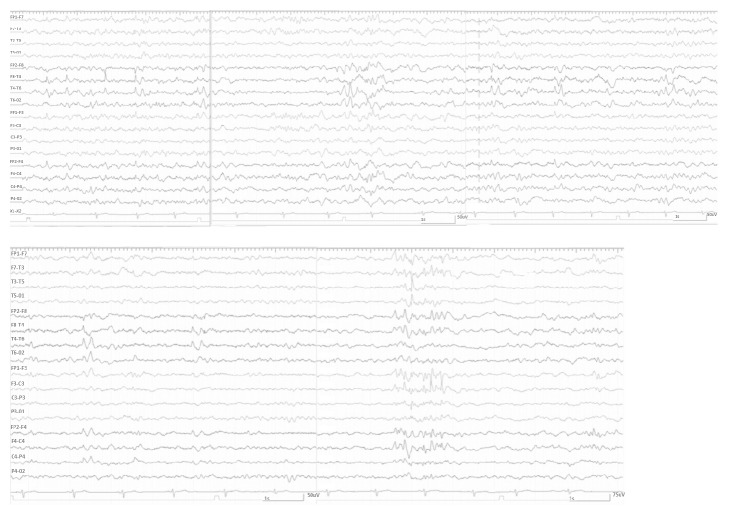
Interictal EEG pattern of the patient.

On neuropsychological examination, the patient was moderately mental retarded but spoke fluently with no apparent dysfunction in all language aspects. Physical and neurological examination (including minimal mental state) was normal. The interictal EEG revealed slowing and sharp waves over the right temporal anterior region (F8) with spreading to the whole temporal and suprasylvian area. Interictal EEG pattern is presented in Figure 1.

**Figure 2:**
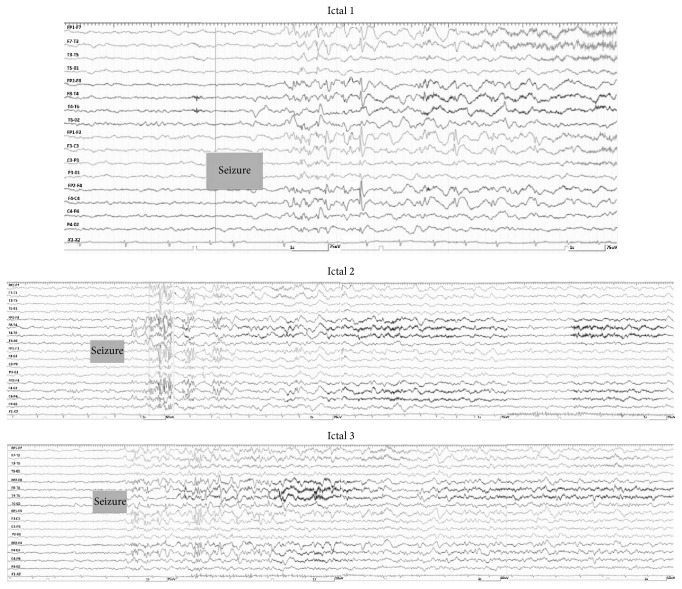
Ictal EEG pattern of the patient during three-seizure attacks.

**Figure 3:**
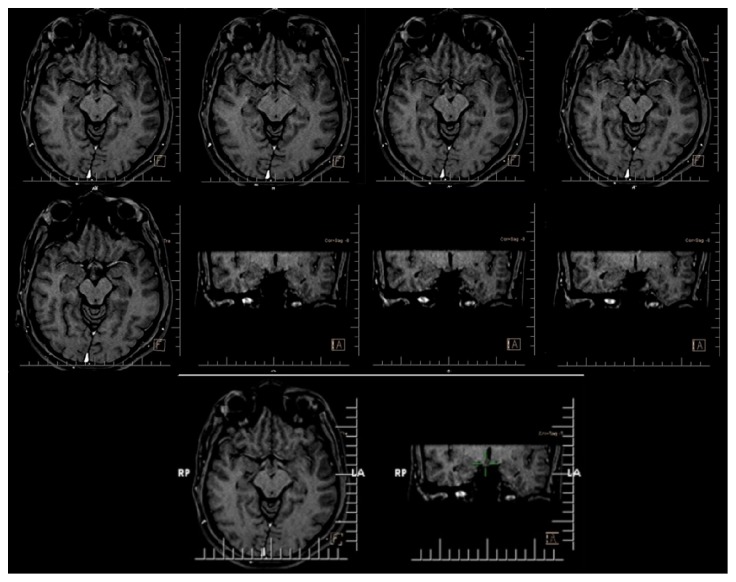
MRI of the patient revealing hypothalamic hamartoma.

During his hospitalization he experienced three different seizures. The first episode presented by tonic spasm for 13 seconds in both hands exactly after he woke up. The second episode presented by hypermotor activity and right deviation of head for 22 seconds with subtle automatism exactly before ictal phase termination. The third episode lasted for 1 minute and 9 seconds with right deviation of head, extension of right hand, and abduction of left hand and was generalized secondarily. He experienced oral automatism in postictal phase. Ictal EEG pattern is illustrated in Figure 2. Magnetic resonance imaging (MRI) demonstrated HH displacing mammillary body (Figure 3). We should note that HH was the only abnormal finding in patients' MRI.

The patient was discharged from hospital after the mentioned evaluations with his current anticonvulsant regimen and was candidate for surgical intervention and removal of the hypothalamic hamartoma; however, the epilepsy surgery was not performed due to lack of patient consent.

## 3. Discussion

Here, we report a case of early onset drug resistant epilepsy that has been misdiagnosed as idiopathic generalized epilepsy for years. After long-term video-EEG monitoring and required examinations and imaging, the diagnosis of hypothalamic hamartoma was proposed for the patient and he got candidate for surgical removal of the tumor.

Generally, HHs are very rare conditions mostly presenting with precocious puberty, gelastic epilepsy, and cognitive difficulties [2, 4]. The very small prevalence of HH causes the condition not to be known and experienced in many centers and many neurologists may encounter only one or two of these cases through their carrier [3–5]. This may cause the disease not to be diagnosed and treated early, like the case we presented.

HHs usually lead to seizures in infancy, typically presenting with gelastic seizures. These seizures are rarely accompanied by altered consciousness in infancy but may present with impairment of consciousness as the patient gets older. Also, autonomic phenomenon, automatisms, epigastric auras, déjà vu, déjà vecu, crying, and motor symptoms may occur [2, 3]. In our case, gelastic seizures were not reported neither in childhood nor later. Although gelastic seizures are known as the hallmark of HH, very small number of patients may present other seizure types such as tonic seizures [6]. Our patient had also this rare presentation of HH. No other epileptogenic source was identified in patients' MRI or EEG and HH was the only finding responsible for his seizures. Deterioration of childhood seizures into complex seizure disorders is reported in several cases of HH before [4], as it was seen in our patient as well.

Developmental delay is a common presentation of HH, especially in patients with early onset seizures [2]. Our patient had history of developmental delay in childhood and he had started having habitual seizures at the age of 6 months. Patients with HH are also known to have some behavioral difficulties, including emotional instability, irritation, agitation, and aggression. Also they may develop attention deficit/hyperactivity disorder, conduct disorder, learning impairment, speech retardation, and anxiety [3, 7]. Our patient had history of learning impairment and speech retardation and as we stated in physical examination he was moderately mentally retarded.

Anticonvulsant drugs, including Primidone, can cause vitamin D insufficiency as an adverse drug reaction [8]. Furthermore, vitamin D insufficiency may be associated with hypocalcemia which can cause seizures, as there are reports on patient that stopped having seizures after treatment of hypocalcemia [9]. Our case had documented vitamin D insufficiency which was substituted at time of diagnosis. Also, he had no report confirming hypocalcemia. The vitamin D insufficiency in our case may be related to taking antiepileptic drugs and we believe it is not related to his seizures.

Medical treatment of these patients usually results in failure [10]. Therefore, surgical intervention has been suggested for them long time ago, and different surgical approaches have been tried based on clinical and imaging findings [10]. To diagnose HH, neurologists must consider typical clinical presentation of this condition, positive findings on patients' history, and evidence of HH on MRI concomitantly. Nevertheless, these cases may be misdiagnosed because of both obsolete symptoms some of them may present and very rare prevalence of this condition [5].

Here we reported a patient with a rare presentation of HH. Our patient had seizure semiology of falling, tonic spasm of limbs, oral automatism, vocalization, and hypermotor activities. He started having habitual seizures at age of 6 months which switched to current seizures few years ago. He also had delayed developmental milestones and was mentally retarded. Although the patient did not experience gelastic seizures as the hallmark of HH, other symptoms could lead the examining neurologist to the diagnosis of HH, which was not reached during 25 years of the disease. The diagnosis might be reached earlier if clinical symptoms, patients' history, EEG, and imaging findings were being investigated more precisely and with having in mind HH as an etiology of drug resistant epilepsy.
